# Correlation between variant call accuracy and quality parameters in comprehensive cancer genomic profiling tests

**DOI:** 10.1016/j.plabm.2024.e00369

**Published:** 2024-02-15

**Authors:** Hideaki Isago, Kousuke Watanabe, Yumiko Satoh, Makoto Kurano

**Affiliations:** Department of Clinical Laboratory, The University of Tokyo Hospital, Tokyo, Japan

**Keywords:** Comprehensive genome profiling tests, NGS, Visual inspection, Assay validation^1^

## Abstract

**Background:**

Comprehensive genomic profiling (CGP) tests have been widely utilized in clinical practice. In this test, the variant list automatically output from the data analysis pipeline often contains false-positive variants, although the correlation between the quality parameters and prevalence of false-positive variants remains unclear.

**Methods:**

We analyzed 125 CGP tests performed in our laboratory. False-positive variants were manually detected via visual inspection. The quality parameters of both wet and dry processes were also analyzed.

**Results:**

Among the 125 tests, 52 (41.6%) required more than one correction of the called variants, and 21 (16.8%) required multiple corrections. A significant correlation was detected between somatic false-positive variants and quality parameters in the wet (ΔΔCq, pre-capture library peak size, pre-capture library DNA amount, capture library peak size, and capture library concentration) and dry processes (total reads, mapping rates, duplication rates, mean depth, and depth coverage). Capture library concentration and mean depth were strong independent predictors of somatic false-positive variants.

**Conclusions:**

We demonstrated a correlation between somatic false-positive variants and quality parameters in the CGP test. This study facilitates gaining a better understanding of CGP test quality management.

## Highlights

• Comprehensive genomic profiling often comprises false-positive variants.
• Somatic false-positive variants and quality parameters were strongly correlated.
• Capture library concentration and mean depth can predict these variants.

## Graphical abstract

**Figure:**
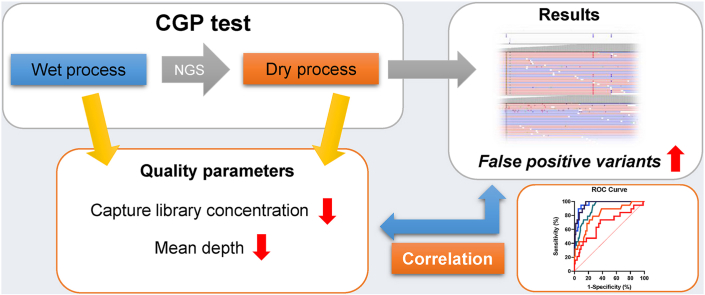

## 1 Introduction

Following the development of next-generation sequencing (NGS) technology, comprehensive genomic profiling (CGP) has been widely used in clinical practice. CGP tests provide information about the cancer genome, including candidate driver mutations, tumor mutation burden, microsatellite instability, and structural variants, which help clinicians select the most effective therapy [1].

In Japan, several CGP tests have been approved by the Pharmaceuticals and Medical Devices Agency and are available under universal health insurance coverage since June 2019 [2]. As of August 2023, more than 60000 CGP tests have been performed and registered in Japan [3]. Among them, OncoGuide™ NCC Oncopanel System (NOP) was developed domestically by the National Cancer Center and Sysmex corporation. NOP is a matched-pair test that uses normal peripheral blood and cancer tissues that to detect somatic variants with high precision and definitively diagnose germline variants [4].

In the CGP test, abundant sequence data is generated via NGS. Bioinformatic pipelines interpret these data as a variant list. As the pipeline output sometimes includes false-positive variants, the laboratory must manually check and correct these variants, a process called visual inspection or manual review [5]. It is a vital process in CGP tests; however, only few hospitals perform visual inspections, as most CGP tests are performed in commercial laboratories. Consequently, the prevalence of false-positive variants and their correlation with the quality parameters have not been described in literature.

At our hospital, we run a genome laboratory equipped with NGS and an insourced NOP test; we perform NOP tests on clinical samples from our hospital. In this study, we report our experience with insourced CGP tests and demonstrate the correlation between quality parameters and occurrence of somatic false-positive variants.

## 2 Material and methods

### 2.1 Data collection and ethical review

Between December 2021 and September 2023, the 125 NOP tests performed at our hospital were included. This study was approved by the Ethical Review Board of the Faculty of Medicine of the University of Tokyo (No. 2021221G). Informed consent was obtained from the patients as opt-out forms on the website.

### 2.2 Clinical sequencing

**Fig. 1:**
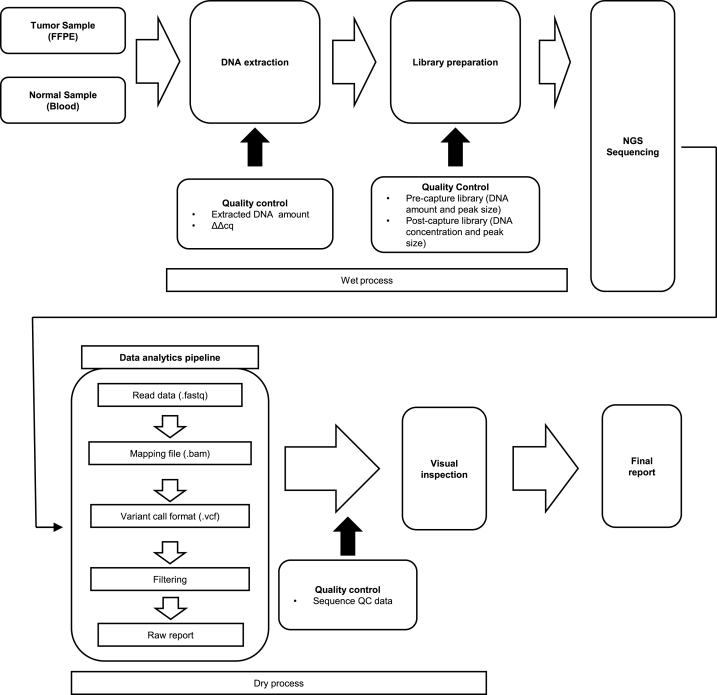
NCC oncopanel test workflow and quality control details.

**Fig. 2:**
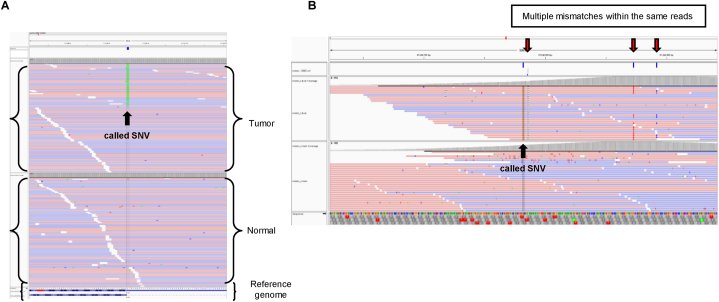
Examples of false-positive variant detection via visual inspection A. Example of a somatic SNV variant in an IGV viewer. B. Example of a false-positive variant. “High discrepancy region” caused by misalignment. Tumor and normal reads shown are from artificial DNA synthesized for quality checks.

All analyses were performed in the International Organization for Standardization 15189-certified laboratory at the University of Tokyo Hospital (Fig. 1). Briefly, genomic DNA was extracted from formalin-fixed paraffin-embedded tumor tissues and peripheral blood. The extracted DNA was subjected to the first quality control, which involved quantification using Qubit Fluorometer (Thermo Fisher Scientific, MA, USA) and tumor DNA degradation assessment using the ΔΔCq method. In the ΔΔCq method, the fragmentation of DNA is evaluated using qPCR with two set of primers of different lengths. ΔCq is calculated as Cq_(long)_ – Cq_(short)_ and ΔΔCq is calculated as ΔCq_(sample)_ - ΔCq_(reference)_. The extracted DNA was then fragmented via sonication and subjected to library preparation, which included end repair, A-tailing, adaptor ligation, polymerase chain reaction amplification, hybridization, and library capture. Peak sizes and DNA amounts in the pre-capture library were measured before hybridization. Peak size and DNA concentration of the post-capture library were measured after library capture. NGS was performed using the NextSeq 550Dx (Illumina, CA, USA) as per manufacturer's instructions. The generated sequence data were analyzed using the OncoGuide™ NCC Oncopanel Analysis Program (Ver 2.01–00 or Ver 2.02–01). BAM and VCF files were used for visual inspection and the raw report from the analysis program was transformed into a final report (Fig. 2).

### 2.3 False-positive variants detection

Prior to visual inspection of clinical tests, the operators (two doctors and one medical technician in our department) received training from the manufacturer for it. The operator's skills were tested and certified by the manufacturer after training via analyzing standard samples.

All somatic and germline variants were inspected manually using the Integrated Genome Viewer (Fig. 2) [6,7]. The call and annotation of false-positive variants were done based on standard operating procedures proposed by Barnell et al. [5]. Two operators reviewed the data independently, and discrepancies of decisions were resolved by discussion and consensus. When variants could not be determined, we sought advice from the bioinformatics division of the NOP manufacturer.

### 2.4 Statistical analysis

Data processing and analysis were performed using GraphPad Prism 9.2.0 software (GraphPad Software, CA, USA). Between-group comparisons were performed using Student's *t*-test. For predicting false-positive variants, the receiver operating characteristic (ROC) curve and the area under it (AUC) were calculated and optimal cut-off points were established. Spearman's rank correlation was used for correlation analysis between quality parameters. Multiple logistic regression analyses were performed to obtain the odds ratios between quality parameters and occurrence of false-positive variants. Model performance was validated using the chi-square test and Hosmer-Lemeshow analyses. The Wald test was used to determine the significance of each variable.

The results were considered significant when the p-value was <0.05. The p-values were described as * for <0.05, ** for <0.01, *** for <0.001, and **** for <0.0001.

## 3 Results

### 3.1 Characteristics of analyzed cases and details of false-positive variants

**Fig. 3:**
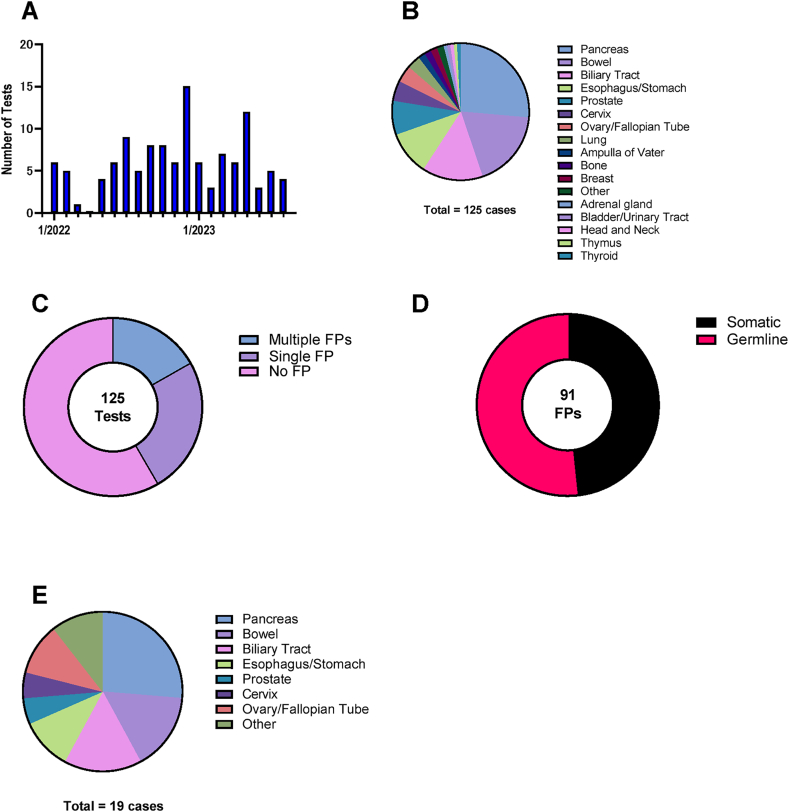
Details of NOP analyzed cases and false-positive variants A. Number of NOP tests per month. B. Distribution of cancer types. C. Prevalence of false-positive variants. D. Origin of false-positive variants. E. Distribution of cancer types of cases with somatic false-positive variants.

From December 2021 to September 2023, 125 cases were analyzed (mean, 5.95 cases per month, Fig. 3A). The cancer type distribution is shown in Fig. 3B. The most frequent cancer type was in the pancreas (26.4%), followed by the bowel (18.4%) and biliary tract (14.4%).

Visual inspection revealed that 52 cases (41.6%) had more than one false-positive variant and 21 (16.8%) required multiple corrections (Fig. 3C). A total of 91 false-positive variants were detected, half of which were somatic (Fig. 3D). The distribution of tumor type in cases with more than one false-positive variant was almost the same as that in the total cases (Fig. 3E).

Details of the false-positive variants are listed in Table 1. In somatic variants, false-positives were observed in several genes among which they were frequently observed in TP53 and NF1. In contrast, in germline variants, a significant number of false-positives were observed for a few specific genes: TSC2, NF1, and VHL. False-positive variants were detected in 9.52% of all somatic variants, whereas germline false-positive variants were detected in 11.38% of all germline variants.

**Table 1:** Details of detected false-positive variants.

Somatic Variants		Germline Variants	
**Gene**	**Number of False-Positives**	**Gene**	**Number of False-Positives**
TP53	18	TSC2	27
NF1	6	NF1	9
ATM	2	VHL	6
BRCA2	2	MLH1	2
ABL1	1	BRCA2	1
APC	1	SMAD4	1
CTNNB1	1	PMS2	1
EGFR	1		
FBXW	1		
JAK3	1		
KDM6A	1		
KIT	1		
MAP2K1	1		
NOTCH2	1		
NTRK2	1		
PALB2	1		
PIK3R1	1		
PTEN	1		
RAF1	1		
RET	1		
Total false-positive variants	44	Total false-positive variants	47
False-posietive variants/total variants	9.52% (44/462)	False-posietive variants/total variants	11.38% (47/413)

The variant tags of false-positive variants			
Somatic Variants		Germline Variants	
Tags	Number of False-Positives	Tags	Number of False-Positives
Low mapping quality	35	Directional	44
Same starts/ends	7	Multiple mismatches	2
Directional	1	Low Variant Frequency	1
False call of amplification^a^	1		

a False amplification calls are not defined in the SOP proposed by Barnell et al.

The annotations of false-positive variants also differed between the somatic and germline variants. In somatic variants, majority were annotated as having “low mapping quality.” In our visual inspection, this type of false-positive call often resulted from inadequate or insufficient read alignment in the mutation detection algorithm for known pathogenic variants in the CGP test. In germline variants, almost all variants were annotated as “directional,” which was assigned when called variants were only found on reads sequenced in either the positive or negative direction. Overall, different types of errors were identified between somatic and germline false-positive variants.

### 3.2 Characteristics of quality parameters in false-positive variants

To identify the factors contributing to false-positive variants, we compared the results of the quality parameters with or without false-positive variants.

**Fig. 4:**
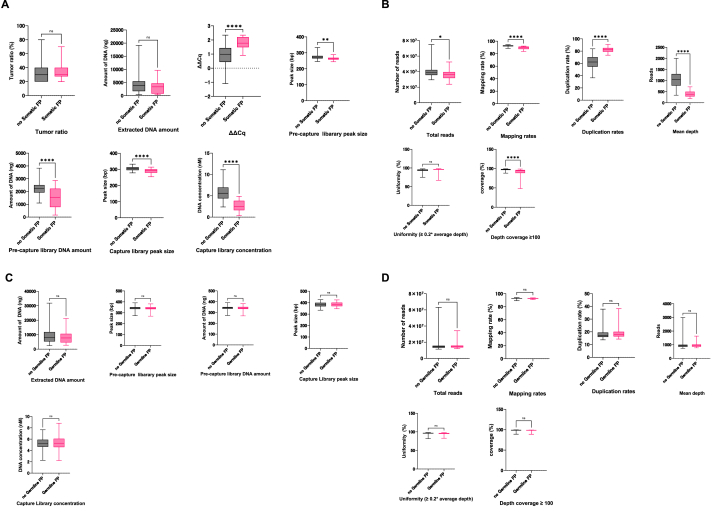
Characteristics of quality parameters in false-positive cases A. Quality parameters before sequencing somatic false-positive variants (wet process). B. Quality parameters after sequencing somatic false-positive variants (dry process). C. Quality parameters before sequencing germline false-positive variants (wet process). D. Quality parameters after sequencing germline false-positive variants (dry process). The p-values were denoted as * for <0.05, ** for <0.01, *** for <0.001, **** for <0.0001.

In wet process quality parameters, cases with somatic false-positive variants showed significantly inferior quality compared to those without false-positive variants in ΔΔCq, pre-capture library peak size, pre-capture library DNA amount, capture library peak size, and capture library concentration (Fig. 4A). For the dry process quality parameters, cases with somatic false-positive variants exhibited significantly inferior quality in total reads, mapping rates, duplication rates, mean depth, and depth coverage (Fig. 4B).

In contrast, no significant differences were observed in quality parameters between cases with and without germline false-positive variants (Fig. 4C and D). Based on these results, we concluded that somatic false-positive variants were strongly associated with the inferior quality of extracted tumor DNA, captured library, and sequencing, whereas germline false-positive variants were generated independent of the quality parameters of wet and dry processes.

### 3.3 Correlation between quality parameters and prediction of somatic false-positive variants

After conducting a univariate analysis, we assessed the predictive performance of each quality parameter.

**Fig. 5:**
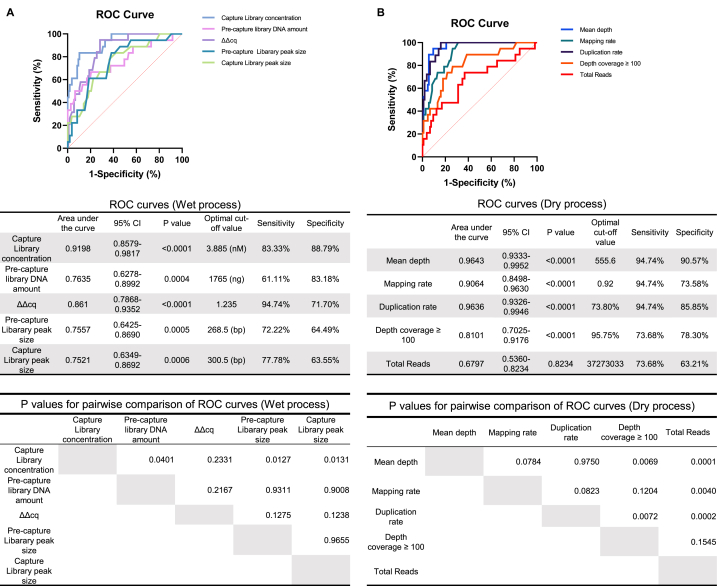
Correlation between quality parameters to predict somatic false-positive variants A. ROC curves of correlation between false-positive variants and quality parameters in the wet process, and p-values for the pairwise comparison of ROC curves. B. ROC curves of correlation between false-positive variants and quality parameters in the dry process, and p-values for the pairwise comparison of ROC curves.

First, we examined the correlation between quality parameters that significantly changed in somatic false-positive variants. The correlation matrix between quality parameters revealed a strong correlation (r ≥ 0.7) between ΔΔCq and duplication rates or tumor mean depth, capture library concentration and duplication rates, and duplication rates and tumor mean depth (Supplementary Fig. 1). To determine the most suitable quality parameter for predicting false-positive variants, we conducted a ROC curve analysis. In the wet process, capture library concentration emerged as the strongest predictor of somatic false-positive variants, followed by ΔΔCq and pre-capture library DNA amount (Fig. 5A). The AUC for capture library concentration in predicting somatic false-positive variants was 0.9198 (95% confidence interval, 0.8579–0.9817), with an optimal cutoff level of 3.885 nM, sensitivity of 83.33%, and specificity of 88.79%. A pairwise comparison of ROC curves showed that capture library concentration is a significantly superior predictor of somatic false positive variants compared to other quality parameters, except ΔΔCq. Multiple logistic regression analysis of quality parameters in the wet process demonstrated that the capture library concentration remained a robust independent predictor of somatic false-positive variant occurrence (Table 2A).

**Table 2:** Results of multiple logistic regression analysis A. Correlation between false-positive variants and quality parameters during wet processing. Model Chi-square test: p < 0.0001, Hosmer-Lemeshow test: p = 0.9744.

Variables	β(coefficient)	Odds Ratio	Odds Ratio 95% CI	p-Value
ΔΔCq	1.299	3.664	0.7377 ～ 23.90	ns
Pre-capture library DNA amount	−0.001049	0.999	0.9973 ～ 1.000	ns
Pre-capture Library peak size	−0.0244	0.9759	0.9056 ～ 1.049	ns
Capture Library peak size	0.0565	1.058	0.9542 ～ 1.141	ns
Capture Library concentration	−1.316	0.2683	0.1194 ～ 0.5035	<0.001

Variables	β(coefficient)	Odds Ratio	Odds Ratio 95% CI	p-Value
Total reads	1.626E-07	1.000	1.000 ～ 1.000	ns
Mapping rates	−0.6153	0.5405	0.2337 ～ 0.9937	ns
Mean depth	−0.01267	0.9874	0.9771 ～ 0.9942	<0.001
Depth coverage ≥ 100	0.06018	1.062	1.8132 ～ 1.264	ns

B. Correlation between false-positive variants and quality parameters during the drying process. Model Chi-square test: p < 0.0001, Hosmer-Lemeshow test: p = 0.9600.

In the dry process, ROC curves showed that mean depth was the most potent predictor of somatic false-positive variants, followed by duplication and mapping rates (Fig. 5B). The AUC for mean depth was 0.9643 (95% confidence interval: 0.9333–0.9952) with an optimal cutoff value of 555.6, sensitivity of 94.74%, and specificity of 90.57%. A pairwise comparison of ROC curves showed that mean depth and duplication rate were significantly superior predictors of somatic false positive variants compared to other quality parameters, except mapping rate. Owing to the strong correlation between mean depth and duplication rates (r > 0.9), duplication rates were excluded from variables for the multiple logistic regression analysis of quality parameters in the dry process (Table 2B). The results showed that mean depth remained an independent predictor of somatic false-positive variants in the dry process.

## 4 Discussion

In this study, we demonstrated that somatic false-positive variants were occasionally observed in CGP tests. To the best of our knowledge, this is the first study to report a correlation between variant calling accuracy and quality parameter results from both wet and dry processes in CGP tests. Although several reports have highlighted the correlation between variant call accuracy and quality parameters [[8], [9], [10]], these studies have primarily focused on sequencing quality parameters. In our clinical genome laboratory, we conducted both wet and dry processes and demonstrated that false-positive variants could be predicted by quality parameters of the wet process in addition to sequencing quality, as reported previously [9]. Estimating the quality of CGP tests before sequencing could be beneficial for clinicians, aiding in their decision to proceed with sequencing and subsequent processes, which are typically the most expensive and time-consuming stages.

A significant portion of somatic false-positive variants were identified as “low mapping quality.” Low mapping quality indicates low confidence in the correct read alignment [11]. Our study revealed that the quality parameters related to library quality, mapping quality, and mean depth served as predictors of somatic false-positive variants.

NOP employs a hybrid capture method for target enrichment that selectively captures the target genomic region using synthesized sets of probes (baits). If the capture process is not optimal, the library may contain low-quality DNA in terms of size, amount, and concentration. The input of a low-quality library inevitably produces low-quality sequence data, which in turn deteriorates quality parameters of the drying process, including mapping rates, duplication rates, depth coverage, and mean depth. Low-quality sequencing data can lead to misalignment or low-confidence mapping of reads, resulting in false-positive somatic variants. This assumption is supported by correlations between the wet and dry quality parameters (Supplementary Fig. 1).

The underlying factors contributing to differences in library quality are unknown. As the formalin-fixing and paraffin-embedding process inevitably deteriorates nucleic acid integrity over time [12], clinical tumor samples exhibit wide variations in DNA quality. Sample storage duration or preservation conditions may also serve as predictors of somatic false-positive variants. Further studies are required to explore these aspects.

In contrast, we found that germline false-positive variants are usually tagged as “directional,” which indicate the occurrence of strand bias on variant supporting reads. We could not identify any predictors of false-positive germline variants among the quality parameters. Strand bias arises from various mechanisms, including sequencing errors and read misalignments. In our study, germline false-positive variants were detected at specific positions in a few genes, suggesting that the genome sequence, characteristics of capture probes, or data analytics pipeline may explain the germline false-positive variants.

The prevention of false-positive variants is difficult. As shown here, false-positive variants are produced in specific genes; hence, reconfiguring program pipelines might decrease the number of false-positive variants. However, it might affect the sensitivity of true variant detection, which inevitably produces false-negative variants. Additionally, as CGP tests are approved by the Pharmaceuticals and Medical Devices Agency in Japan, it is prohibited to make modifications to them in medical institutes. In conclusion, visual inspection by trained personal would remain a vital process in preventing the occurrence of false-positive variants for now (Fig. 1).

Our study has several limitations. First, our data were limited to false-positive variants identified in NOP tests. Only a few insurance-covered CGP tests are available in Japan. We conducted the NOP tests in our clinical laboratory because other tests were permitted only in domestic or foreign commercial laboratories. The methods used for analysis are assumed to be relatively consistent among CGP tests, as they usually adopt a combination of capture-library methods and short-read NGS. The application of our results to other CGP tests remains unverified.

Second, this was a retrospective analysis of CGP tests and we did not evaluate the application and efficacy of our findings. Predicting the somatic false-positive variant occurrence may be useful as an indicator for added meticulousness to visual inspection of called variants and a basis for deciding the continuation of CGP tests; however, further prospective studies are warranted to confirm this hypothesis.

In conclusion, our study elucidates the correlation between wet and dry quality parameters and prevalence of somatic false-positive variants in CGP tests. This study contributes to a better understanding of CGP test quality management.

## Role of the funding source

This research did not receive any specific grant from funding agencies in the public, commercial, or not-for-profit sectors.

## CRediT authorship contribution statement

**Hideaki Isago:** Writing – review & editing, Writing – original draft, Resources, Project administration, Methodology, Investigation, Formal analysis, Conceptualization. **Kousuke Watanabe:** Writing – review & editing, Resources, Project administration, Methodology, Investigation, Formal analysis. **Yumiko Satoh:** Writing – review & editing, Resources, Investigation, Conceptualization. **Makoto Kurano:** Writing – review & editing, Supervision, Conceptualization.

## Declaration of competing interest

The authors declare that they have no known competing financial interests or personal relationships that could have appeared to influence the work reported in this paper.

## Data Availability

The data that has been used is confidential.
