# Supplementary figures and images for: Correlation between variant call accuracy and quality parameters in comprehensive cancer genomic profiling tests
Source: Pract Lab Med. 2024 Feb 15;39:e00369. doi: 10.1016/j.plabm.2024.e00369 (PMC10884978; doi:10.1016/j.plabm.2024.e00369)

## Slide 1
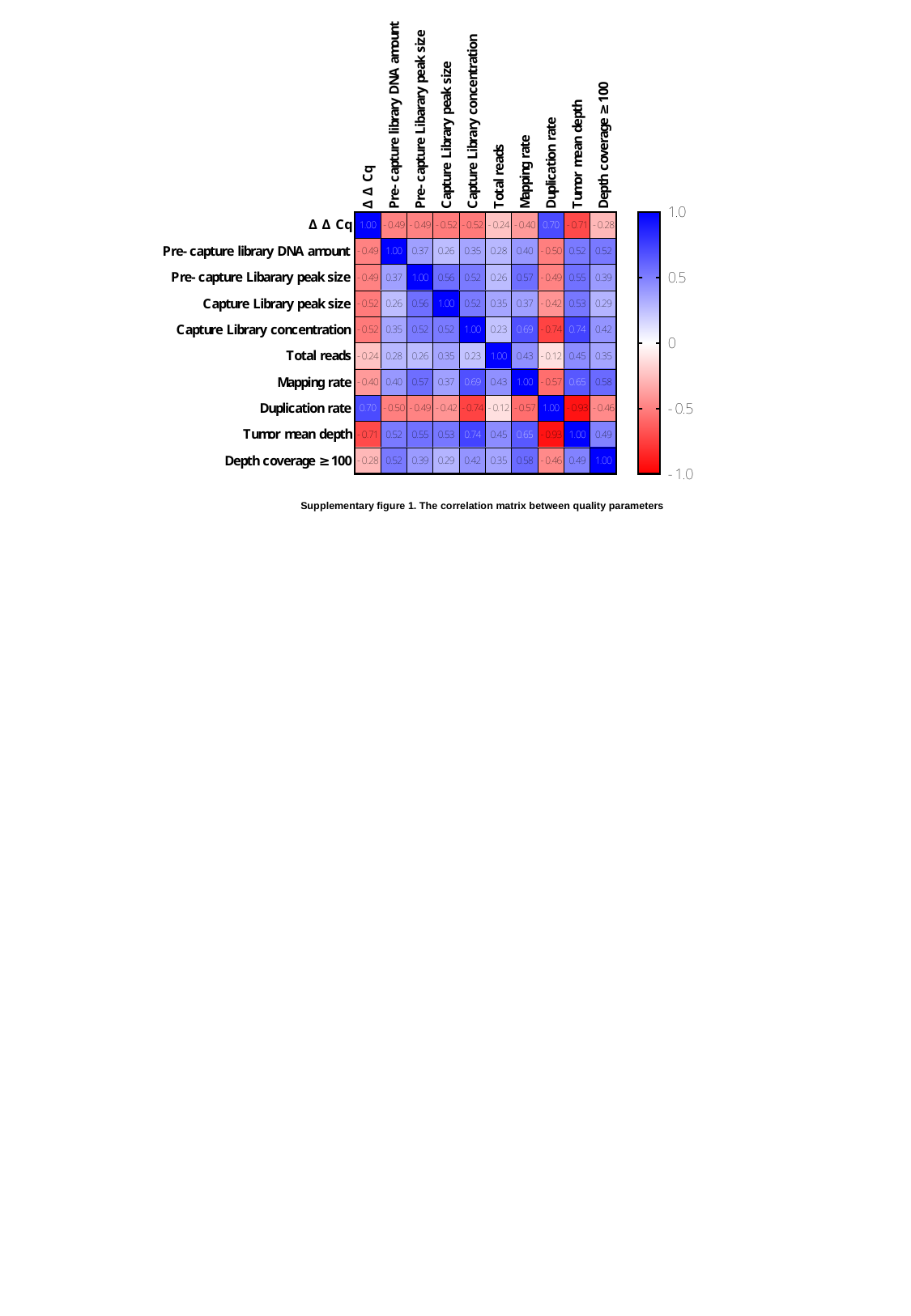

Supplementary figure 1. The correlation matrix between quality parameters

Supplement: Multimedia component 1 [file mmc1.pptx]
